# Synthesis and application of a new antibacterial surfactant from apricot kernel oil

**DOI:** 10.1038/s41598-023-48404-x

**Published:** 2023-12-06

**Authors:** Hanaa M. Soliman

**Affiliations:** Fats and Oils Department, Institute of Food Industries and Nutrition, National Research Centre, Dokki, Giza Egypt

**Keywords:** Chemistry, Organic chemistry, Surface chemistry, Chemical synthesis

## Abstract

Food emulsifier are mostly prepared from a lipophilic lipid tail with a hydrophilic sugar head. In this study, the lipophilic tail was obtained from apricot kernels, which are food waste, and the hydrophilic head was gluconic acid instead of sugar, in order to draw attention to the non-cyclic poly hydroxyl compounds. Thus, oleic acid of apricot kernel was used as the lipophilic moiety of the prepared surfactant. So, apricot kernel was grinned and dried, oil was extracted using soxhlet apparatus, Physical and chemical parameters and fatty acids composition of the extracted oil had been determined. The extracted oil was then hydrolyzed into glycerol and a mixture of free fatty acids. The fatty acids mixture was separated. Then, oleic acid was extracted individually in pure form using supercritical CO_2_ extractor, it was then confirmed according to its melting point, Gas chromatography–mass spectrometry (GC–MS) after esterification, elemental analysis, Proton nuclear magnetic resonance (H^1^NMR), and mass spectrometry (MS) to detect the corresponding molecular ion peak. The pure individual oleic acid was converted to hydroxy stearic acid, which was then converted to an amphiphilic compound (surfactant) via esterification reaction with the hydrophilic gluconic acid, and afforded a new surfactant known as 2,3,4,5-tetrahydroxy-6-((9-((-2,3,4,5,6-pentahydroxyhexanoyl) oxy)octadecanoyl) oxy)hexanoic acid or stearyl gluconate for simplification. The structures elucidation of all synthesized compound was established according to elemental analysis and spectral data (Fourier transform infrared IR, ^1^H NMR, ^13^C NMR and MS). Moreover, the prepared compound was tasted for its antibacterial activity, and showed good activities against some types of bacteria. The surface-active properties, foamability, foaming stability and emulsion stability of stearyl gluconate were studied and compared with the properties of the well-known surfactant sucrose stearate, and it was clear that, the activity of stearyl gluconate as a surfactant was higher than that of sucrose stearate. Moreover, establishment of safety of this compound was performed using albino rats by acute oral toxicity and kidney and liver functions of these mice. On the other hand, the prepared surfactant was used in the production of low fat—free cholesterol mayonnaise as egg replacer. Texture properties and the sensory evaluation of the prepared mayonnaise showed that the properties were improved by using the new prepared surfactant. Thus, the prepared gluconyl stearate can be used as a safe food additive.

Surfactants is a shortened word for surface active agents. These are a class of chemical compounds known as amphiphiles. It consists of a polar head attached to a non-polar tail. This amphiphilic property of surfactant molecules causes affinity for adsorption at the interfaces (a link of any two immiscible phases) thus reducing the surface tension, where the hydrophilic polar head of surfactant molecules strongly prefers interaction with polar entities and the hydrophobic non-polar tail strongly prefers interaction with hydrophobic entities^1,2^.

Surfactants are usually categorized according to the chemical nature of their heads into four categories known as nonionic, anionic, cationic and amphoteric surfactants^3^, each category has its own applications. Surfactants are widely used in our lives, they are an essential ingredient in personal care products, household cleaners, pharmaceuticals, oil recovery, food processing, and nanotechnologies^4^.

The food industry is highly interested with the production of non-ionic surfactants of carbohydrate fatty acid esters as they are biodegradable, tasteless, odourless, nontoxic, antimicrobial, and insecticidal compounds^5,6^. Therefore, they are used as food additives where they are widely used to improve the flavour, texture and shelf life of food, typically they are used as foaming agent for cream, ice-cream, latte and coffee, cakes, desserts and shortening ,as spreading agent for cheese, chocolate, margarine and butter, as emulsifying agent for dressing salads and blended fat, as gelling agent, as film forming and antimicrobial agents, as modifying agent for dough, as control modifier to the fat’s crystallization for milk products those containing acid or salt and as anti- swelling and anti-foaming agent for tofu production^7–11^.

Usually, carbohydrate fatty acid esters are prepared via chemical or enzymatic esterification reaction between mono or disaccharides as hydrophilic head with mono or diglyceride as lipophilic tail and sometimes the lipophilic tail is replaced with fatty acid^12^. According to the degree of un saturation, chain length, linearity, branching degree, cyclization and number of hydroxyl groups surface tension properties are varied^13^.

On the other hand, mayonnaise is one of the most favorite sauces around the world, it is a white to off-white, cream or sauce, commonly used on sandwiches, hamburgers, salads, hot dogs, French fries, seafood, and more. Traditional mayonnaise is oil in water emulsion containing 70–80% oil. Therefore, it is considered unhealthy due to its high oil content, where eating large amounts of fat increases risk of obesity, some types of cancer, cardiovascular disease and high blood pressure^14^. Thus, attention has been shifted towards the production of low-fat mayonnaise via decreasing of the dispersed phase and increasing of the continues phase, and consequently, poor texture, flavour, appearance, stability, and mouth feel were observed. Then, so many studies have been carried out by scientist in order to enhance properties of low-fat mayonnaise. Some of these studies had used deferent classes of emulsifiers.

## Experiment

### Instruments

The infrared spectra were recorded for potassium bromide on a Pye Unicam Sp 3–300 Shimadzu FT IR 8101 PC infrared spectrophotometer. The NMR spectra were recorded on a Varian Mercury VX-300 NMR spectrometer. ^1^H spectra were run at 300 MHz. Chemical shifts are quoted in δ and were related to that of the solvents. The mass spectra were recorded on a Shimadzu GCMS-QP-1000EX mass spectrome-ters at 70 e.V. Elemental analyses was carried out at the Micro-analytical Center of Cairo University. Supercritical CO_2_ instrument used in the present study was applied Sep-arations. Inc., Allentown, USA, model no.7071. Metrohm 679 Rancimat (Metrohm AG, Herisau, Switzerland). Brookfield DVIII model digital rheometer.

### Material and reagents

#### Chemicals

Sulfuric acid, ethanol, diethyl ether and gluconic acid were purchased from the British Drug House (BDH). All the other chemicals used in the current study were obtained from Sigma Chemical Company (England, Landon, Ltd., Pools).

#### Plant material and seeds collection and preparation

Optimum maturity Apricot fruits were obtained during the harvesting season (May 2022) from Nubariya town, Egypt. The plant was approved to be collected, its taxonomic identity was verified by Prof. Dr. Shaban D. Abou-Hussein at National Center, Giza, Egypt. Moreover, a voucher herbarium specimen was deposited in publicly available herbarium at the National Research Center. The fruits were washed with running water, dried with a towel, the pulp and peels were separated, the seeds were cleaned, the seeds shells were opened with a strong nutcracker, and the kernels were collected. apricot kernels were grinded using grinder (Moulinex -LM241- France), and dried overnight at 40 °C, and moisture content was determined. The dried grinded kernels were kept at −18 °C until use.

All method comply with relevant institutional, national, and international guidelines and legislation.

The study is reported in accordance with ARRIVE guidelines. The experimental protocol was approved by Medical Research Ethics Committee—National Research Centre (Approval 211055022).

### Apricot kernel oil extraction

Apricot kernel (200 g) were grinned and dried overnight at 40 °C, moisture content was determined. Oil was extracted with diethyl ether (500 ml) in a Soxhlet Apparatus at a condensation rate of 5 or 6 points per second for 6 h. The solvent was then evaporated to dryness using a rotary evaporator, and the obtained Apricot kernel oil was weighted and kept for analysis ^15^.

### Quality parameters of the extracted oil

#### Refractive index (RI)

The refractive indexes (RI) of oil samples were determined using a refractometer (Rudolph model J157 at 20˚C), each test was performed three repetitions^16^.

#### Acid value (AV)

A mixture of absolute ethanol and diethyl ether (1:1 v/v) was carefully neutralized with 0.10 N potassium hydroxide solution using 1% phenolphthalein indicator. About 5 g of the tested samples were dissolved in 50 ml neutralized ethanol-diethyl ether solvent and titrated with 0.10 N potassium hydroxide with constant shaking until a pink color persisted for 15 s.^16^. Free fatty acids were calculated as % of oleic acid.

$$ FFA\% \,as\,oleic\,acid = S \times 0.0282 \times 100/W $$

where *S* = titration (ml), *W* = weight of the oil (g).

#### Iodine value (IV)

The Iodine value of the tested samples was determined using Hanus method^16^. Briefly, about 0.25 g of tested sample was weighted into 500 ml glass stopper flask and dissolved in 20 ml chloroform. 25 ml Hanus iodine (13.2 g pure I_2_ in 1 L CH_3_COOH) solution was added and let stand 30 min in dark. 10 ml 15% KI solution was added shacked thoroughly and then100 ml freshly boiled and cooled H2O was added. I_2_ was titrated with 0.10 N sodium thiosulphate solution with constant shaking until yellow solution turns almost colorless. A few drops of 1% starch solution as indicator was added and titration was continued until blue entirely disappears with shaking vigorously to release all iodine from CHCl_3_. A blank was performed omitting the oil, and the iodine value was calculated as grams of iodine per 100 g of oil.

$$ Iodine\,value = \left( {B - S} \right) \times N \times 12.69/W $$

where *B* = ml Na_2_S_2_O_3_ solution required for blank; *S* = ml Na_2_S_2_O_3_ solution required for test sample; *N* = Normality of Na_2_S_2_O_3_ solution, and *W* = weight of sample in g.

#### Peroxide value (PV)

Tow g from tested oil were weighted in flask with ground-glass cap. 250–300 ml, 10 ml of chloroform were added and shacked for 10 min, 15 ml of glacial acetic acid, and 2 g of sodium bicarbonate NaHCO_3_ were added, after stirring, 1 ml of a saturated solution of KI was added and shacked for 1 min and kept in dark place for 5 min, immediately. After the specified time, 75 ml distilled water, then 0.5 ml of starch solution were added, the resulting solution was titrated with a solution of Na_2_S_2_O_3_ (0.01 N) to the disappearance of the blue color, the blank was carried on the same procedures but without sample^16^. A peroxide value of the tested oil given by the equation:

$$ PV = \left[ {\left( {V1 - V2} \right)*N*1000} \right]/W\left[ {meq.O_{2} /kg} \right] $$

where *V1* = volume of sodium thiosulfate solution consumed in the titration of sample (ml), *V2* = volume of sodium thiosulfate consumed in the titration of the blank (ml), *N* = the normality of sodium thiosulfate, *W* = weight of fat taken to denote (g).

#### Determination of unsaponifiable matter content

Saponification value of the tested oil samples was determined^16^. Briefly, 5 g of the tested sample was weighted into 250–300 ml conical flask, and 50 ml alcoholic KOH solution (35–40 g KOH were dissolved in 20 ml water and diluted to one liter with alcohol, 95%) was added. Flask was connected with air condenser, boiled until fat was completely saponified (~ 30 min), cooled and titrated with 0.5 M HCl using phenolphthalein. Saponification value was calculated by the following formula:

$$ Saponification\,value = 28.05\left( {B - S} \right)/W $$

where *B* = ml HCl required for blank; *S* = ml HCl required for test sample; *W* = weight of the sample in g.

#### Total polar materials (TPM)

TPM were determined in oil samples and measured by column chromatographic^17,18^.

#### Polymer content (PC)

PC was determined^19^. One gram of oil was added to methanol (125 ml) containing 1% H_2_SO_4_. The mixture was boiled under a reflux condenser for 2h and cooled to room temperature. The methanol insoluble were filtered and washed with methanol until no sulphuric acid remained. The washed insoluble polymers were dissolved in petroleum ether (25 ml) and transferred to a pre-weighed flask. The solvent was then evaporated under a stream of nitrogen and the flask was again weighted.

#### Viscosity

Apparent viscosity was measured^20^. Where computerised Brookfield viscometer model RV-DV (Laboratories. Inc. USA) was used.

### Fatty acids composition

The fatty acids composition of the extracted fat was determined by GC–MS after their esterification^16^. Fatty acids were esterified into their corresponding methyl esters by shaking a solution of fatty acids (0.1 g) in heptane (2 ml) with methanolic potassium hydroxide solution (0.2 ml, 2 N). The fatty acid methyl esters were identified using a gas chromatograph. Nitrogen flow rate was 0.6 ml /min., hydrogen and air-flow rates were 45 and 450 ml/min, respectively. The oven temperature was isothermally heated 195 °C. The injector and the detector temperatures were 230 °C and 250 °C, respectively. Fatty acid methyl esters were identified by comparing their retention times with known fatty acid standard mixture. Peak areas were automatically computed by an integrator.

### Oil hydrolysis

Apricot kernel oil (92.4 g, 46.2% of kernel weight) was hydrolyzed using a high-pressure reactor and distilled water (250 ml) for 3h in at 250 °C and 2 MPa. Then, the reaction was left to cool to room temperature in order to separate the fatty layer from the non-fatty one. The separated fatty layer was, dried over anhydrous sodium sulfate and filtered off. The formation of free fatty acids was confirmed using ^1^H NMR spectrum and TLC^21^. Where, plates (20 × 20) were coated with a slurry of silica gel (60g) in water (15g /kg), left to dry, then it was activated at 110 °C for 1.0 h. Standard spot of known fatty acid and another spot of oil (individually) were spotted on the activated thin layer plates, at the baseline (2 cm from the bottom). The fatty acids mixture of the hydrolyzed fat was also spotted on the same line. The developing solvent consisted of n-hexane, diethyl ether and acetic acid at a volumetric ratio of 80: 20: 1, respectively. The developing jar was lined on three sides with filter paper wetted with the same developing solvent. The plates were developed till the solvent reached the front line (15 cm from the start line). The spots of different components separated by TLC were then visualized by iodine vapor. The fatty acids were considered formed when the withdrawn sample showed only one spot with no tail, and with the rate of flow similar to that of the known fatty acid spot but not the oil spot.

### Individual extraction of oleic acid

Oleic acid was extracted individually in a pure form from the fatty layer (80.44 g) of the extracted Apricot kernel oil using a supercritical CO_2_ extractor at 28.0 MPa, 313 K. The obtained oleic acid (48.83 g, 98.6% of the actual oleic acid content in the apricot oil) was confirmed according to its elemental analysis, melting points, GC–MS and mass spectra (MS) of its methyl ester which detect the corresponding molecular ion peak^21^.

Yield (48.83 g, 98.6%), (pale yellow liquid), m.p. (13 °C), IR (KBr) ν_max_/cm^–1^: 3015–2955 (= CH), 2923–2845 (CH- aliphatic), 2785 (OH), 1732 (C=O), 1618 (C=C). ^1^H NMR (CDCl_3_): δ 0.92 (t, 3H, J = 6.9Hz, 18-CH_3_), 1.26–1.33 (m, 20H, 4–7 and 12–17-CH_2_), 1.56(m, 2H, 3-CH_2_), 2.06 (m, 4H, 8,11-CH_2_), 2.20 (t, 2H, J = 7.0 Hz, 2-CH_2_), 5.35–5.42(m,2H, 9,10-CH),11.3 (s, D_2_O-exchangble, 1H).^13^C NMR (DMSO—d_6_): δ14.0, 22.7, 24.6, 27.6, 29.0, 29.3, 29.5, 29.7, 29.9, 31.9, 34.7, 130.5, 179.2. MS *(m/z)*: (295M^+^), 156, 126, 97, 59, 71, 55. For C_18_H_34_O_2_ (282.47): Calcd: C, 76.54; H, 12.13%. Found: C, 76.6; H, 11.9%.

### Organic synthesis

#### Synthesis of 9-Hydroxyoctadecanoic acid

Hydroxy octadecanoic acid was prepared^22^ via sulphating oleic acid, followed by hydrolysis. Where, sulfuric acid (15 ml, 96%) was added dropwise to a stirred cooled oleic acid (20 g, 0.07 mol) in an ice bath, after the addition was completed, the stirring was continued in the ice bath for another 30 min. After that, water (100 ml) was quickly poured over the mixture, and stirred under reflux for one hour, then, the reaction was left to cool to room temperature. By using of a separating funnel, the aqueous layer was removed, while the fatty layer was washed for several time with water. Alcoholic KOH solution (50 ml) was added to the fatty layer and stirred under reflux for 8 h. The crude product was collected after evaporation of the alcohol by means of a rotatory evaporator, then, it was neutralized with dilute sulfuric acid. Finally, the formed hydroxy stearic acid was washed with hot water till neutralization, and recrystallized from ethanol by standing over night at room temperature.

Yield (18.32 g, 86%), (mp. 58–59 °C), (white wax-like), IR (KBr) ν_max_/cm^–1^: 3624 (OH alcoholic), 2920–2842 (CH- aliphatic), 2791 (OH acidic), 1735 (C = O). ^1^H NMR (CDCl_3_): δ 0.91 (t, 3H, J = 7.0 Hz, 18-CH_3_), 1.26–1.33 (m, 22H, 4–8 and 12–17-CH_2_), 1.57(m, 2H, 3-CH_2_), 2.06 (m, 4H, 9,11-CH_2_), 2.1 (t, 2H, J = 7.1 Hz, 2-CH_2_), 3.5(m, 1H, 10-CH), 4.8 (s, D_2_O-exchangble, 1H), 10.97 (s, D_2_O-exchangble, 1H).^13^C NMR (DMSO—d_6_): δ14.1, 22.7, 24.7, 25.6, 29.0, 29.3, 29.6, 29.9, 32.0, 34.0, 37.7, 72.33, 179.1. MS *(m/z)*: (299 M^+^), 214, 128, 113, 59, 57. For C_18_H_36_O_3_ (300.84): Calcd: C, 71.95; H, 12.08%. Found: C, 71.66; H, 12.28%.

#### Synthesis of 2,3,4,5-tetrahydroxy-6-((9-((-2,3,4,5,6-pentahydroxyhexanoyl)oxy)octadecanoyl) oxy)hexanoic acid or ( stearyl gluconate)

To stirred refluxed acetone (150 ml), potassium hydroxide was gradually added until the PH reaches 10.5. the mixture was left to cool to room temperature, and filtered off. The filtrate was stirred, gluconic acid (19.62 g, 0.1 mol) was gradually added, followed by addition of hydroxy stearic acid (10 g, 0.033 mol). The reaction mixture was stirred under reflux for six hours, then, the reaction was left to cool. Acetone was evaporated using a rotatory evaporator. The remainder (potassium hydroxide, unreacted hydroxy stearic acid, unreacted gluconic acid, stearyl gluconate, coupling product of hydroxy stearic acid with itself and the formed dimers and polymers of gluconic acid coupling) was washed with ice/cooled water for several times, in order to get rid of potassium hydroxide, unreacted gluconic acid and the formed dimers and polymers of gluconic acid, (where the very cooled water could not dissolve stearyl gluconate as it was found to be semi solid at low temperature). Then, the residue (unreacted hydroxy stearic acid, coupling product of hydroxy stearic acid with itself and stearyl gluconate) was washed with boiled water, where hydroxy stearic acid and the coupling product of hydroxy stearic acid with itself are in soluble in hot water, so, it could be separated and removed. The hot aquatic solution was then cooled in a refrigerator overnight, the formed precipitate of stearyl gluconate was filtered off and dried overnight under vacuum.

Yield (19.88 g, 91%), mp. 68–69 °C. IR (KBr) ν_max_/cm^–1^:, 3611(carboxylic OH) 3324–3377 (9 alcoholic OH), 2919–2842 (CH- aliphatic), 1732, 1751 (2C = O), 1211,1230 (2 C-O).^1^H NMR (CDCl_3_): δ 10.88(s, D_2_O-exchangble, 1H), 5.18(2 s, D_2_O-exchangble, 1H), 4.53(s, D_2_O-exchangble, 1H), 4,49(2 s, D_2_O-exchangble, 1H), 4.39(4 s, D_2_O-exchangble, 1H), 4.33(t, 2H), 4.11(s,1H), 3.99 (d, 2H), 4.05(q, 1H), 3.77(t, 2H), 3.60(d, 2H), 3.51(q,1H), 3.37(t, 2H), 2.33(t, 2H, J = 6.9 Hz, 2-CH_2_ ), 1.59(m, 2H, 3-CH_2_), 1,49(m, 4H, 9,11-CH_2_), 1.25–1.33 (m, 22H, 4–8 and 12–17-CH_2_), 0.91 (t, 3H, J = 6.9 Hz, 18-CH_3_). ^13^C NMR (DMSO—d_6_): δ 14.0, 22.7, 25.1, 25.3, 29.1, 29.8, 32.2, 33.6, 35.3, 64.0, 66.1, 69.2, 70.3, 71.6, 72.4, 72.9, 73.4, 75.12, 171.7, 175.6, 177.3. For C_30_H_56_O_15_ (294.475): Calcd: C, 54.86; H, 8.59%. Found: C, 54.85; H, 8.37%.

### Anti bacterial activity

The prepared compounds (100 µg/ml) were tested in vitro for antibacterial activity against four bacterial species (two gram positive and two gram negative) namely *Staphylococcus Epiderm, Enterococus fesalis, Enterobacter cloacae,* and *Flavo-Bacterium,* using disk diffusion method^23^, for the antibacterial activity of each compound in diethyl ether as solvent. Inhibition zone diameter (IZD), in mm/mg compound was tested and taken as the criterion for antimicrobial activity. The antibiotics *Vacomycin—rifampin, Penicillin G, Gentamycin—cephpalosporin—cefotaxime Imipenem* and* Normal flora Natpathegen,* were used as references to evaluate the potency of the tested compounds under the same condition.

### HLB calculation

According to Griffin^25^, the HLB values of non-ionic surfactants can be calculated using the following formula:

$$ HLB = 20\left( {MH/M} \right). $$

where MH is the molar mass of the hydrophilic moiety and M is that of the whole surfactant molecule.

### Surface active properties

Surface active properties of the prepared surfactant have been measured using surface tensiometer (POWEREACH JK99B, Shanghai Zhongchen Digital Technical Apparatus Co., Ltd., China).

#### Oil–water interfacial tension γo/w

Sunflower oil had been purified before use, where, sunflower oil (500 g) was stirred with deionized water (50 ml) for 1h at room temperature, then it was centrifuged, the aqueous phase which contained free fatty acid was discarded. Activated carbon was added to the oily layer (25%, w/w) and the mixture was left over night. Afterwards, the pure filtrate was obtained via centrifugation followed by filtration. Sunflower oil was deemed acceptable only when the interfacial tension against the deionized water remained constantly above 25 mN/m for 1 h.^25^.

Then, sunflower oil–water interfacial tensions were measured according to Du Noüy method^26^. Where solutions of the prepared stearyl gluconate and sucrose stearate at 25 ◦C (0.05 wt. %) individually, in deionized water (0.01% w/v) were prepared. Then, a clean and heated to redness platinum ring of known geometry was suspended horizontally, the ring was dipped in the tested liquid and pulled again afterwards. The maximum force needed to pull the ring through the interface is expressed as the interfacial tension.

#### Determination of critical micelle concentration (CMC) by air–water surface tension method

Surface tensions of stearyl gluconate solutions at different concentrations were measured with a surface tensiometer equipped with a Wilhelm plate^27^ at 25 °C, and compared to that of the well-known sucrose stearate. The platinum plate of known geometry was thoroughly washed and heated to redness before each measurement. Where, a homogenous aqueous surfactant solution (50 ml) was placed individually in Petri dishes, the platinum plate was vertically hanged on a suspension device of the tensiometer, then its lower edge was brought into contact with the liquid surface. The sample vessel was lifted until the plate dipped into the tested liquid. The maximum force needed to pull the plate through the surface is expressed as the surface tension. A plot of surface tension versus concentration of surfactant was produced. The CMC was determined from the inflection point.

### Foamability and foaming stability

The method used for measuring foaming power and foam stability was carried out^28^. Where, an individual aqueous solutions of sucrose stearate and stearyl gluconate (10 ml, 0.25 wt./v) at 25 °C were placed in 50 ml graduated cylinder. The solution height of each sample was recorded (H_0_, cm). Then, each solution was mixed using Ultra-Turrax homogenizer T25 at 13,500 rpm for 2 min, the total height (H_1_, cm) and the foam height (H_2_, cm) were determined immediately. After standing for 45 min, the foam height (H_3_, cm) was recorded at 25°C. The foamability and foaming stability were calculated using the following equations:

$$ {\text{Foamability }}\left( \% \right) \, = \, \left\{ {\left( {{\text{H}}_{{1}} - {\text{H}}_{0} } \right) \, /{\text{ H}}_{0} } \right\}{\text{ x 1}}00 $$

$$ {\text{Foaming stability }}\left( \% \right) \, = \, \left( {{\text{H}}_{{3}} /{\text{H}}_{{2}} } \right){\text{ x 1}}00 $$

### Emulsion stability

According to Griffin^24^ the HLB values of the prepared stearyl gluconate was found to be 11.4, thus, it could only apply to oil-in-water emulsions.

So, an individual solutions of sucrose stearate and stearyl gluconate in distilled water (60 ml, 0.5% w/v) were mixed using an Ultra-Turrax homogenizer T25 at 9500 rpm for 1 min. The dispersed phase (oil phase (40 ml)) was slowly added, the mixture was homogenized at 13,500 rpm for 2 min^29^, the formed emulsion was transferred to a 100-ml graduated cylinder and held at 25 °C. The volume of separated oil phase was measured at 0, 4, 8, 12, 16, 20 and 24 h. Oil separation Percent over a given period was calculated according to the following formula:

$$ {\text{Separation }}\% = \, ({\text{Volume of separated oil phase }}/{\text{ Total Volume of oil}}) \times {1}00 $$

### Acute toxicity test and Liver and kidney function tests:

Albino mice of 22-25g weight were housed individually under standard conditions (12-h light/dark cycle; 25 ± 3 °C temperature; 35–60 relative humidity). The study is reported in accordance with ARRIVE guidelines. The experimental protocol was approved by Medical Research Ethics Committee—National Research Centre (Approval 1195852022). Thirty animals were divided into six groups (5 animals/group), one group was used as control and was fed on standard mice feed only, and the others were given a mixture of oral doses of the prepared stearyl gluconate with different concentrations and standard mice feed^30^.

The liver and kidney function of those Albino mice were tested to improve the safety of stearyl gluconate as a food additive.

Thus, Alanine transaminase (ALT), Aspartate transaminase (AST), Alkaline phosphates (ALP), Creatinine, and Urea were determined spectrophotometrically^31,32^.

Statistical significance between the control and experimental data were subjected to one-way analysis of variance (ANOVA) followed by Tukey post hoc test, using Microsoft Excel 2016 MSO (16.0.10361.20002) 32-bit.

### Uses of the prepared gluconyl stearate as an egg replacer for mayonnaise production

The control low fat mayonnaise was prepared according to method described by Hala Amin^14^ with slight modification. The recipe contained the following ingredients in percentage (w/w): sunflower oil 45, water 38, vinegar (5% w/v) acetic acid)) 10, dried egg yolk 3, sugar 1.0, salt 1.0 and mustard 2. On the other hand, dried egg yolk was replaced with the previously prepared Stearyl gluconate in order to get a free cholesterol low fat mayonnaise. Where, egg yolk or stearyl gluconate was individually mixed with water and vinegar using a homogenizer mixer (Model 584, Tefal, France) in high speed for 3 min. Then, salt, sugar and mustard were added, and homogenization process was continued. Finally, oil was slowly added with continues stirring at 6000 rpm for 3 min. until the emulsion system was established, followed by stirring at 2000 rpm for another 4 min. finally, the prepared samples were kept in the refrigerator at 4 °C until the physicochemical and sensory analysis.

#### Apparent viscosity of the prepared mayonnaise

Apparent viscosity of samples was measured^33^. Where computerised Brookfield viscometer model RV-DV (Laboratories. Inc. USA) was used. Samples viscosity at different temperatures (4, 25, 40 °C) were measured using spindle No. 7 at rotating speed of 100 rpm.,

#### Color measurement

Mayonnaise samples were measured for color^34^ in the L*, a*, b* system using Hunter Lab color analyzer (Hunterlab Colour Flex EZ, USA).

#### Texture

Texture measurements were determined^14^ with the TA.XT2i Texture Analyzer (Stable Micro Systems Ltd, Surrey, UK) with a 5 kg load cell. Back extrusion cell with 35 mm diameter compression disc was used. The samples were carefully transferred into acrylic cylindrical containers (50 mm internal diameter and 75 mm height) to a depth of 55 mm. One cycle was applied, at a constant crosshead velocity of 1 mm/s, to a sample depth of 40 mm, and then returned. From the resulting force–time curve, the values for texture attributes, i.e. firmness, adhesiveness and cohesiveness were obtained using the texture expert for window version 1 equipment software.

### Panel test

A total of 10 non-trained panelists were recruited. The samples were spread on a toast pieces and labeled using two random digits. The sensory evaluation of the mayonnaises was studied using a structured scale from 1 to 10 (1 = lowest score and 10 = highest score) for assessing the general appearance, taste, texture, and acceptability^35^. the results were expressed as percentage (%). All the panelists provided written informed consent.

### Ethical approval

The experiments were conducted following the ethical guidelines for investigations in laboratory animals and complied with the National Institutes of Health guide for the care and use of laboratory animals (NIH Publications No. 8023, revised 1978). All applicable international, national, and/or institutional guidelines for the care and use of animals were followed.

## Results and discussions

### Apricot kernel oil extraction

To facilitate the extraction of apricot kernel oil, the moisture must be removed, and thus the apricot kernel was grinded in order to increase its surface area which facilitates the dehydration process. Moisture content was found to be (9.65g, 4.82% of the kernel weight). Then, extraction of the oil was performed using soxhlet apparatus and diethyl ether. The oil yield was (92g, 46.2% of the kernel weight).

### Quality parameters and fatty acid profile of the extracted apricot kernel oil

The extracted apricot kernel oil was characterized as mentioned in Table 1. Where its refractive index, acid value, peroxide value, iodine value and saponification number were 1.473, 2.24 (mg KOH/g oil), 5.10 (mEq/Kg oil), 103.3 (grams of I_2_ /100 g oil), 201.2 (mg KOH/g oil) and 5.3 (%) respectively.

**Table 1:** Physicochemical properties of the extracted Apricot kernel oil.

Parameter	Apricot kernel oil
Refractive index at 25°C	1.473 ± 0.05
Acid value (mg KOH /g oil)	2.24 ± 0.03
Peroxide value (mEq/Kg oil)	5.10 ± 0.02
Iodine value (grams of I_2_ /100 g oil)	103.3 ± 0.91
Saponification number (mg KOH/g oil)	201.2 ± 2.35
Polar contents (%)	5.3 ± 0.02

Data are expressed as mean ± SD values given represent means of three determinations.

Apricot kernels can be considered as a rich source of fatty acids^34^, that play a substantial role in the production of oleochemicals. According to the GC–MS scan after esterification it was found to consists of 93.29% unsaturated fatty acid, those acids are considered as beneficial fats, where they enhance each of blood cholesterol levels, heart rhythms, and inflammation, in addition to so many vital reactions in human body. The predominant fatty acids were oleic acid (C18:1) and Linoleic acid (C18:2), those represent 70.30 and 22.02% respectively. While the dominant were Palmitic acid (C16:0) Palmitoleic acid (C16:1), Stearic acid (C18:0) and Lenolenic acid (C_18:3_), those represent 5.12, 0.59, 1.2 and 0.38% respectively as illustrated in Table 2. The significant levels of fatty acids were generally consistent with those found in previous research^34^. Thus, apricot kernel oil can be considered as a very rich source of oleic acid.

**Table 2:** Fatty acid composition of the extracted Apricot kernel oil.

Fatty acid	Relative amount (%)
Palmitic acid (C16:0)	5.12 ± 0.34
Palmitoleic acid (C16:1)	0.59 ± 0.005
Stearic acid (C18:0)	1.2 ± 0.03
Oleic acid (C18:1)	70.3 ± 0.39
Lenoleic acid (C18:2)	22.02 ± 0.24
Lenolenic acid (C18:3)	0.38 ± 0.022
Others	0.39
Saturated fatty acid (%)	6.32
Unsaturated fatty acid (%)	93.29

Data are expressed as mean ± SD values given represent means of three determinations.

### Oil hydrolysis

The oil consists mainly of a triacylglycerol molecule, which is an ester of glycerol and three fatty acid molecules. Therefore, it can be easily hydrolyzed in a high-pressure reactor to produce glycerol and fatty acid mixture. Thus, the previously extracted apricot kernel oil (92.4 g, 46.2% of kernel weight) was hydrolyzed and gave free fatty acids mixture (80.44g, 87.05% of the extracted oil) and glycerol. The free fatty acids formation was established according to the TLC which showed a unique spot with no tail and with the rate of flow (Rf value) similar to that of known fatty acid but not the oil. Moreover the _1_H NMR spectrum of the formed fatty acids reflects a carboxylic hydrogen signal at δ10.33.

### Individual extraction of oleic acid

From the free fatty acids mixture (80.44g, 87.05% of the extracted oil), A pure oleic acid was separated individually (48.83 g, 98.6% of the actual oleic acid content in the apricot oil) using CO_2_ extractor at 28.0 MPa and 313 K. Where CO_2_ polarity in its critical condition is very sensitive to word temperature and pressure. The separated oleic acid was then confirmed according to its elemental analysis, melting point (13 °C), GC–MS and mass spectra of its corresponding methyl ester which reflect a molecular ion peak (295 M^+^).

### Organic synthesis

**Scheme 1:**
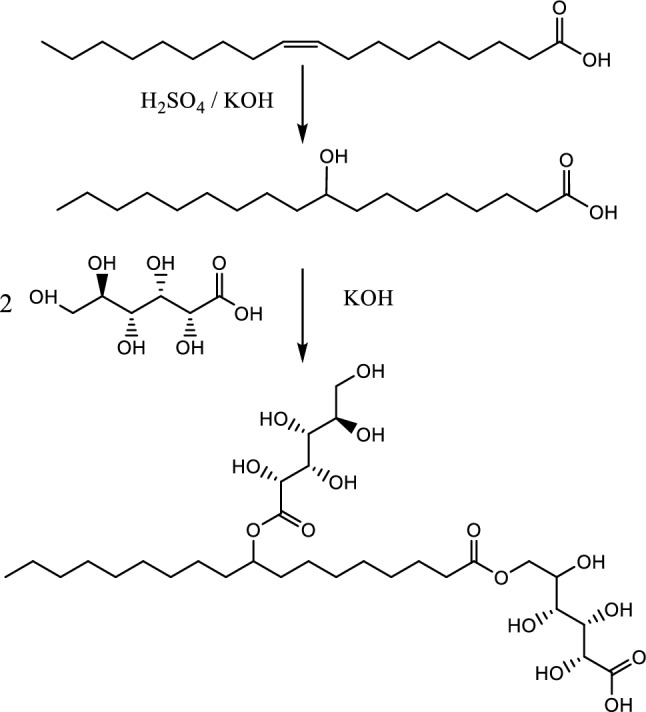
(2*R*,3*S*,4*R*)-2,3,4,5-Tetrahydroxy-6-((9-(((2*R*,3*S*,4*R*,5*R*)-2,3,4,5,6-pentahydroxyhexanoyl)oxy)octadecanoyl)oxy)hexanoic acid.

The new surfactant {2,3,4,5-tetrahydroxy-6-((-10-((2,3,4,5,6-pentahydroxyhexanoyl)oxy)octadecanoyl) oxy)hexanoic acid} was prepared via two steps, the first is conversion of oleic acid into 10 hydroxy stearic acid, and the second is reaction of 10 hydroxy stearic acid with two molecules of gluconic acid, as in Scheme 1.

#### Synthesis of 9-Hydroxyoctadecanoic acid

The pure extracted oleic acid was sulfonated with conc. H_2_SO_4,_ then hydrolyzed with KOH to afford 9-Hydroxyoctadecanoic acid. Thus, the IR spectrum of the prepared compound showed no absorption band at 1618 cm^−1^ which means disappearance C = C function and appearance of an absorption band at 3624 cm^-1^ that indicates presence of alcoholic OH function. On the other hand, its ^1^H NMR spectrum showed two D_2_O-exchangeable signals at 4.8 and 10.97 corresponding to alcoholic and acidic OH groups respectively. Moreover, its mass spectrum revealed a peak at *m/z* 299 (M^+^) corresponding to its molecular ion.

### Synthesis of 2,3,4,5-Tetrahydroxy-6-((9-((-2,3,4,5,6-pentahydroxyhexanoyl)oxy)octadecanoyl) oxy)hexanoic acid or (stearyl gluconate)

Gluconic acid was esterified with 10-hydroxy stearic acid in a strong alkaline medium in order to prevent gluconic acid cyclization into the corresponding glucono-delta-lactone^35^. The formed product was confirmed according to its chemical analysis and spectral data. Where, its IR spectrum showed nine absorption bands at 3324–3377 cm^−1^ due to nine alcoholic OH functions, and other absorption appeared at 3611 cm^−1^ caused by an acidic OH function. The ^1^H NMR spectrum of the same product revealed the appearance of ten D_2_O exchangeable signals related to one carboxylic OH and nine alcoholic OH functions.

### Antibacterial activity

Sucrose stearate showed antimicrobial activities against some gram positive and some gram-negative bacteria^36,37^. This activity was related to the sugar moiety which rupture the bacterial cell membrane^38^. And the variation in their activities are related to their concentrations and the acidity or basicity of the medium^39^.

On the other hand, Table 3 illustrates antibacterial activity results of stearyl gluconate in diethyl ether (100 µg/ml). The inhibition zone diameter (IZD) range between 2 and 10 mm indicates low activity, and it was referred as + , while, inhibition zone diameter (IZD) range between 11 and 24 mm reflects moderate activity, and it was referred as +  + , the inhibition zone diameter (IZD) range between 25 and 35 is corresponding to high activity and it was referred as +  +  + . The study is reported in accordance with ARRIVE guidelines. The experimental protocol was approve by Medical Research Ethics Committee—National Research Centre (Approval 31105512022).

**Table 3:** Antibacterial activity of the stearyl gluconate.

Bacteria		Inhibition Zone Diameter (IZD)	References
Gram + ve	Staphylococcus epiderm	+ + + ve	Vacomycin rifampin + + + ve
	Enterococus fesalis	+ + ve	Penicillin G + + + ve
Gram − ve	Enterobacter. cloacae	+ + + ve	Gentamycin cephpalosporin cefotaxime Imipenem + + + ve
	Flavobacterium	+ + + ve	Normal flora Natpathegen + + + ve

Data are expressed as mean of three determinations.

### Evaluation of the prepared compound as a surfactant

#### HLB calculation

According to Griffin, the HLB values of the prepared 2,3,4,5-tetrahydroxy-6-((9-((-2,3,4,5,6-pentahydroxyhexanoyl) oxy)octadecanoyl) oxy)hexanoic acid was found to be 11.4, and consequently its preferred to be used as oil in water emulsifier.

#### Surface active properties

Surface tension is caused by the intermolecular van der waals force, that drawing the liquid molecules together, or liquid molecules with the other molecules in contact with it (solid, or gas). This force reduces the liquid surface area and consequently increases the surface tension. When surfactant molecules are added they will occupy the positions between the surface film-forming molecules, by forming a new attraction force between the surfactant molecules and the surface film-forming molecules, and thus the intermolecular attraction force between the surface film-forming molecules decreases, and thus the surface area increases and the surface tension decreases. On the other hand, the surface tension energy is approximately equal to the energy required to remove surface film-forming molecules per unit area. Thus, surface active properties of the prepared surfactant have been measured using surface tensiometer.

##### Oil–water interfacial tension γo/*w*

For accurate measurement of oil–water interfacial tension, the oil should be purified, where, presence of free fatty acids and / or polar compounds with in the oil causes lowering of the interfacial tension^40^.

Fatty acids composition of the sunflower oil is illustrated in Table 4. And its physicochemical parameters before and after purification were determined as shown in Table 5.

**Table 4:** Fatty acid composition of Sunflower Oil.

Fatty acid	Relative amount (%)
Palmitic acid (C_16:0_)	6.2 ± 0.04
Palmitoleic acid (C_16:1_)	0.31 ± 0.002
Stearic acid (C_18:0_)	3.2 ± 0.04
Oleic acid (C_18:1_)	35.51 ± 0.34
Lenoleic acid (C_18:2_)	52.18 ± 0.22
Lenolenic acid (C_18:3_)	0.33 ± 0.005
Arachidic acid (C_20:0_)	0.31 ± 0.004
Eicosenoic acid (C_20:1_)	0.28 ± 0.004
Behenic acid (C_22:0_)	0.89 ± 0.003
Lignoceric acid (C_24:0_)	0.30 ± 0.005
Others	0.49
Saturated fatty acid (%)	10.6
unsaturated fatty acid (%)	88.91

Data are expressed as mean ± SD values given represent means of three determinations.

**Table 5:** Physicochemical properties of Sunflower oil.

Parameter	Sunflower oil	
	Before purification	After purification
Refractive index at 25 °C	1.4664 ± 0.02	1.4666 ± 0.03
Acid value (as oleic acid)	0.82 ± 0.01	0.01 ± 0.001
Peroxide value (meq./kgoil)	3.17 ± 0.02	3.19 ± 0.02
Iodine value (Hanus)	134.4 ± 1.20	133.3 ± 1.88
Saponification number	195.7 ± 1.95	188.6 ± 1.44
Polar contents (%)	1.3 ± 0.06	0.0 ± 0.0

Data are expressed as mean ± SD values given represent means of three determinations.

**Figure 1:**
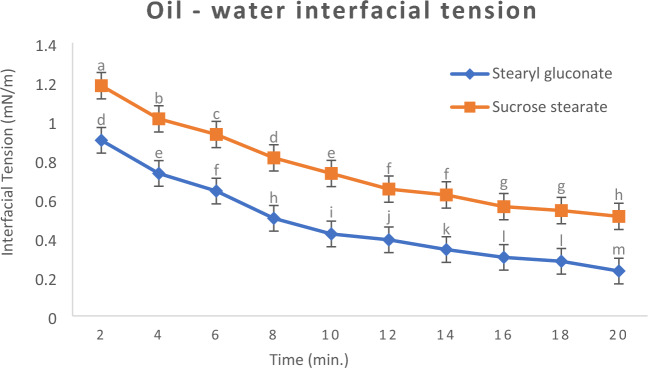
Interfacial tension decay profiles at 25 °C of 0.05 wt. % individual aqueous solutions of Stearyl gluconate and Sucrose stearate. Data are expressed as mean ± SD values given represent means of three determinations.

Oil–water interfacial property of the newly prepared stearyl gluconate was statistically significant (*P* ≤ 0.05) when compared to that of the well-known surfactant sucrose stearate. The new prepared surfactant caused more decreases in the oil–water interfacial. Where, as shown in Fig. 1, the latter is more polar due to the presence of more hydroxyl groups, therefore, it further diffuses between the molecules forming the water surface layer, thus, it reduces the attractive force between them, which causes more increases in the interfacial area and more decreases in the interfacial tension. And this agrees with that reported by Jotam Bergfreund^41^.

##### Determination of critical micelle concentration (CMC) by air–water surface tension method

Surfactant molecules perform their function by diffusion between molecules forming a water surface film. When the water surface film become saturated with surfactant molecules, any more addition of surfactant will not affect surface tension, as they fall into the bulk solution to form aggregates with tails facing inwards away from the water, known as Micelles. The concentration of surfactant at which micelles begin to form is known as the critical micelle concentration (CMC). And this is a property of a surfactant in a given solvent at a given temperature. It could be calculated from the break in the surface tension versus surfactant concentration plots at a known temperature.

**Figure 2:**
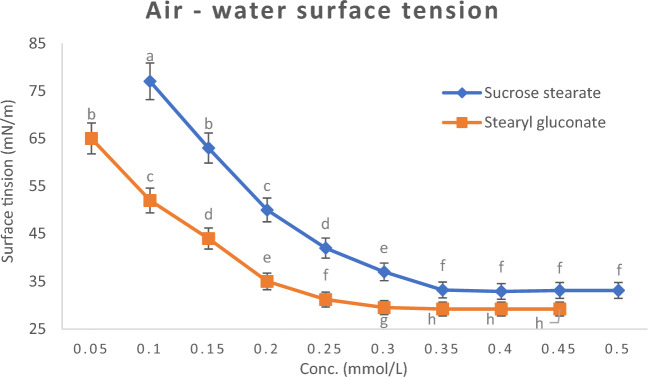
Surface tension versus concentration plots at 25 °C of Stearyl gluconate and Sucrose stearate solutions. Data are expressed as mean ± SD values given represent means of three determinations.

Air–water surface tension property of the newly prepared stearyl gluconate was statistically significant (*P* ≤ 0.05) when compared to that of sucrose stearate as shown in Fig. 2. The particle size of the new prepared surfactant is greater than that of sucrose stearate, therefore, fewer molecules of the newly prepared surfactant diffuses with the molecules that make up the water surface film, and consequently, lower critical micelle concentration is observed. On the other hand, the newly prepared stearyl gluconate is more polar due to the presence of more hydroxyl groups, therefore, it further diffuses between the molecules forming the water surface film, thus, it reduces the attractive force between them, which causes more increases in the surface area and more decreases in the surface tension. And this agrees with that reported by Bronisław Ja ´nczuk^42^.

#### Foamability and foaming stability

Bubbles are formed by mixing a liquid such as water with a gas under pressure or agitation. Once that pressure is removed or loosened, the gas rushes out, forming bubbles that congregate to form foam^39^. The properties of these bubbles differ according to the type of the used gas and surfactant. The phenomenon of surface tension provides an explanation for the foam formation, where surfactant greatly reduces the surface tension force, and thus it becomes possible to make bubbles of large surfaces with a small amount of liquid.

**Figure 3:**
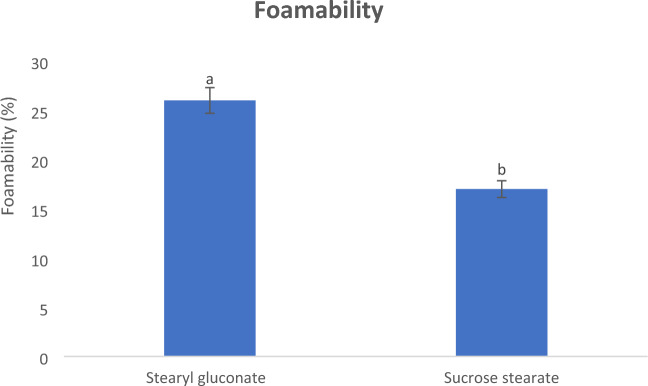
Foaming power of 0.25 mmol/L aqueous solutions at 25 °C of Stearyl gluconate and Sucrose stearate. Data are expressed as mean ± SD values given represent means of three determinations.

**Figure 4:**
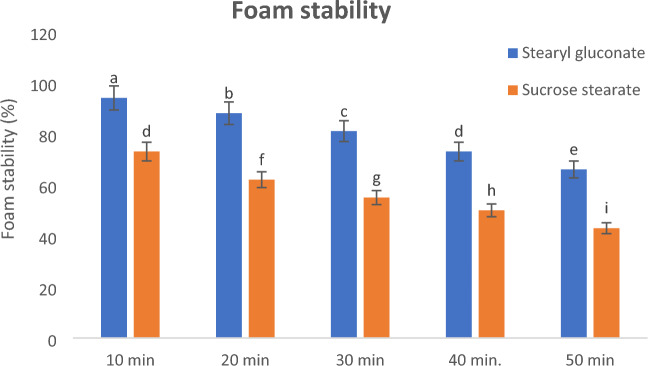
Foam stability of 0.25 mmol/L aqueous solutions at 25 °C of stearyl gluconate and sucrose stearate. Data are expressed as mean ± SD values given represent means of three determinations.

Foamability and foaming stability of the newly prepared stearyl gluconate were examined and compared to those of sucrose stearate as shown in Figs. 3 and 4. The results showed that the foaming ability and foam stability of stearyl gluconates were better than those of sucrose stearate and it was significantly different (*P* ≤ 0.05), and this may be due to its higher efficiency to reduce the surface tension.

#### Emulsion stability

**Figure 5:**
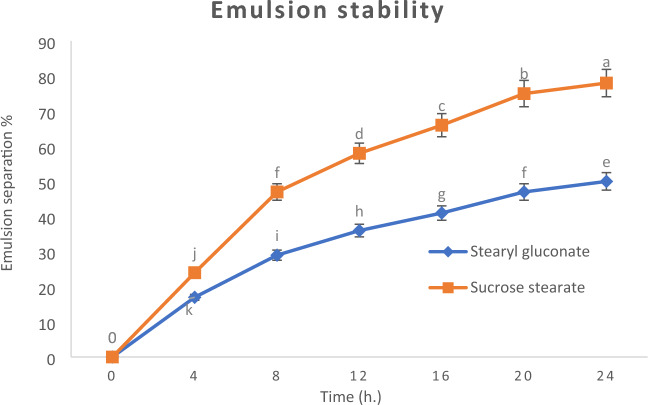
O/W emulsion stability at 25 °C with 0.25mmol/L of stearyl gluconate and Sucrose stearate. Data are expressed as mean ± SD values given represent means of three determinations.

A stable dispersion of two or more immiscible liquids held in suspension by an emulsifier is known as emulsion. And, the emulsion is said to be stable when this stable dispersion doesn’t change with time. So, emulsion stability of the newly prepared stearyl gluconate were examined and compared to that of sucrose stearate and shows significance difference (*P* ≤ 0.05). According to Griffin, the HLB values of stearyl gluconate was found to be 11.4, thus, it is favourable to be applied to oil-in-water emulsions. The results showed that, stearyl gluconate caused higher emulsion stability as shown in Fig. 5.

### Acute toxicity test and liver and kidney functions tests:

The safety of the newly prepared stearyl gluconate was validated based on acute toxicity test and Liver and kidney functions tests. And the results are illustrated in Tables 6 and 7.

**Table 6:** Acute oral lethal toxicity of stearyl gluconate.

Groups	Dose (mmol/kg)	No. of animals/group	No. of dead animals
1	3	5	0
2	6	5	0
3	9	5	0
4	12	5	0
5	15	5	0

**Table 7:** Liver and kidney function tests of rats feed on stearyl gluconate at different concentrations.

Parameter groups	ALT (U/L)	AST (U/L)	ALP (U/L)	Urea (mg/dL)	Creatinine (mg/dL)
Control	21.38^f^ ± 0.15	36.41^d^ ± 0.23	136.62^e^ ± 0.32	33.62^f^ ± 0.15	0.74^c^ ± 0.004
5g/L	21.47^e^ ± 0.18	36.56^d^ ± 0.24	137.55^d^ ± 0.35	34.28^e^ ± 0.17	0.74^c^ ± 0.005
7g/L	21.79^d^ ± 0.17	36.93^c^ ± 0.18	138.44^c^ ± 0.43	34.60^d^ ± 0.17	0.75^b^ ± 0.004
9g/L	22.24^c^ ± 0.17	37.66^b^ ± 0.25	139.57^b^ ± 0.38	34.96^c^ ± 0.20	0.75^b^ ± 0.004
11g/L	22.53^b^ ± 0.16	37.82^b^ ± 0.16	140.79^a^ ± 0.27	35.38^b^ ± 0.19	0.77^a^ ± 0.006
13g/L	22.79^a^ ± 0.17	38.25^a^ ± 0.21	140.98^a^ ± 0.35	35.96^a^ ± 0.18	0.77^a^ ± 0.005

Data are expressed as mean ± SD values given represent means of three determinations.

For each column the common averages in the same letter have no significant differences between them.

The liver and kidney function tests of rats fed the prepared stearyl gluconate at various concentrations are illustrated at Table 2. Analysis of variance test showed that there were statistically significant differences between the different concentrations of liver enzymes (ALT (U/L) AST (U/L) ALP (U/L)). Moreover, there are significant differences between urea (mg/dL) while there are no significant differences in creatinine enzyme (mg/dL). Thus, increasing the concentration of stearyl gluconate resulted in acceptable increases in liver and kidney function, while still within safe limits.

### Uses of the newly prepared stearyl gluconate as an egg replacer for mayonnaise production

The newly prepared stearyl gluconate was found to be a safe and suitable oil-in-water emulsifier. Thus, it can be promising to be used in the production of low-fat free-cholesterol mayonnaise. Therefore, two mayonnaise samples were prepared, one being the control sample where egg was used as an emulsifier, while for the other sample stearyl gluconate was used as an egg substitute emulsifier.

#### Apparent viscosity of the prepared mayonnaise

Viscosity can be defined as fluid's resistance to flow, and in case of emulsion it is strictly related to the close packing of the dispersed oil droplets as they interact with one another in the matrix (the electrostatic force &/or the friction force). The closer the droplets, the higher the viscosity and vis versa^43,44^.

**Figure 6:**
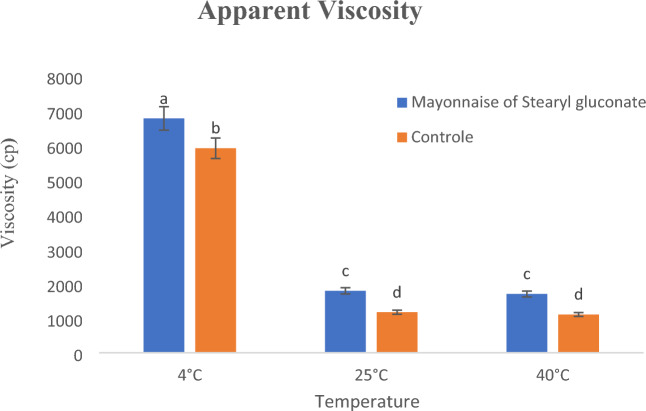
Apparent viscosity of the prepared mayonnaise samples. Data are expressed as mean ± SD values given represent means of three determinations.

There are statistically significant differences (*P* < 0.05) between the viscosity of the two prepared mayonnaise samples, where the use of the newly prepared stearyl gluconate as an emulsifier for the production of mayonnaise leads to a higher viscosity than that resulting from the use of eggs as shown in Fig. 6, and this may be related to the stronger intermolecular attraction force due to the presence of the stearyl gluconate which contains more hydroxyl functions, and consequently formation of more hydrogen bonds (more attraction force). Moreover, ricing the temperature up was found to decrease the mayonnaise viscosity, and this is regarded to the increase of the oil droplet kinetic energy that facilitates its flow and consequently decrease its viscosity.

#### Color measurement

Color is an important indicator of product quality, and is affected by the types and proportions of its ingredients^45^. The light to dark level of the product (in the case of an emulsion) is affected by the number and size of the suspended particles. Since mayonnaise is an oil in water emulsion, the suspended oil droplets act as a spherical refraction surface, so, when light rays fall on those droplets, rays will be refracted in so many directions, and those refracted rays overlap causing the formation of white color. Therefore, by increasing the number of suspended oil droplets and their surface areas, the whiteness increases, and vice versa. So, Hunter Lab color analyzer was used to measure the color of the two prepared mayonnaise samples. This analyzer measures the sample level from light to dark (L*), red to green (a*, where a positive number indicates red and a negative number indicates green) and yellow to blue (b*, where a positive number indicates yellow and a negative number indicates blue).

**Figure 7:**
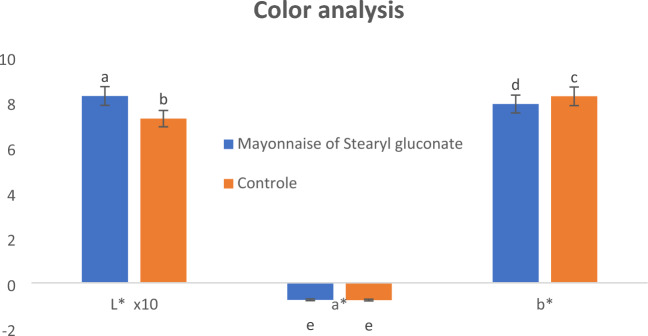
Colour measurements of the prepared mayonnaise. Data are expressed as mean ± SD values given represent means of three determinations.

Figure 7 shows colour measurements of the two prepared mayonnaise samples. The L* values of mayonnaise prepared using stearyl gluconate as an emulsifier were significantly (*P* ≤ 0.05) higher than those of the control sample, this indicates a larger refractive surface area due to the large number and/or large size of the suspended oil droplet, and thus, it represents the efficiency of the used emulsifier to suspend the oil droplets in water. While the a* values showed non-significant differences (*P* > 0.05) between the two prepared mayonnaise samples, the negative denomination for a* values indicates the presence of a green pigment that may be related to the chlorophyll pigment in the used oil. There are significant differences (*P* ≤ 0.05) between the two values of b * for the two prepared samples, and their positive designation indicates the presence of yellow colour, the increase of this yellow colour in the control sample may be related to the presence of carotenoids egg yolk^46^.

#### Texture

**Figure 8:**
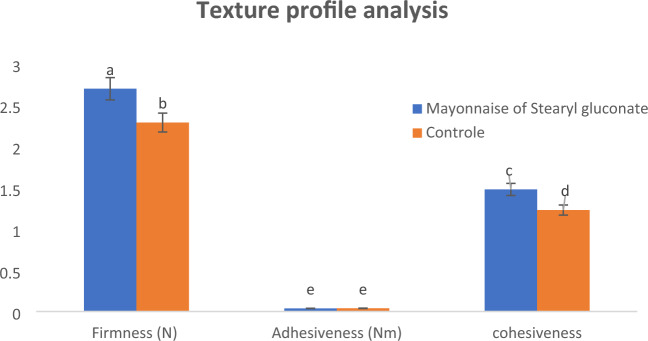
Texture profile analysis of the prepared mayonnaise. Data are expressed as mean ± SD values given represent means of three determinations.

The texture profile of an emulsion is strongly related to the intermolecular force between dispersed phase molecules and each other, and the intermolecular force between molecules of dispersed phase and molecules of continues phase. A texture analyser was used to determine the Firmness, cohesiveness and adhesiveness for the tow prepared mayonnaise samples as illustrated in Fig. 8. The firmness (resistant to externally applied force) of mayonnaise prepared using stearyl gluconate as an emulsifier was significantly (*P* ≤ 0.05) higher than that of the control sample. This indicates the more coalescence of oil droplets by using of stearyl gluconate. On the other hand, cohesiveness of mayonnaise prepared using stearyl gluconate as an emulsifier was significantly (*P* ≤ 0.05) higher than that of the control sample, this means molecules are more tightly together due to the more intermolecular attraction forces between them, thus causing the bulk property of liquid resisting separation^47,48^. Moreover, adhesiveness of the two prepared mayonnaise samples was non-significant (*P* > 0.05).

#### Sensory evaluation

Sensory evaluation of the prepared mayonnaise showed that, uses of stearyl gluconate as an emulsifier for mayonnaise production enhanced its properties as shown in Table 8.

**Table 8:** Sensory evaluation of the prepared mayonnaise.

Parameters	Control mayonnaise	Mayonnaise of stearyl gluconate
Appearance	8.2 ± 0.14	8.9 ± 0.33
Taste	8.5 ± 0.23	8.8 ± 0.21
Color	8.9 ± 0.30	8.2 ± 0.22
Flavor	8.0 ± 0.43	8.5 ± 0.21
Texture	7.2 ± 0.44	8.8 ± 0.21
Overall acceptability	8.16 ± 0.50	8.64 ± 0.55

Data are expressed as mean ± SD values given represent means of three determinations.

## Conclusion

The newly prepared stearyl gluconate showed higher surface activity than the well-known emulsifier sucrose stearate, this may be attributed to the non-cyclic structure of the hydrophilic moiety, which causes less restrictions for the OH functions and consequently facilitate the formation of hydrogen bonds and consequently the attachment to the polar molecules. Moreover, Due to the presence of the long fatty tail with in the new synthesized Antibacterial surfactant, its assumed that, the new synthesized compound can easily penetrate the lipoprotein cell membrane of bacteria and move inside, then, hydrogen bonds are formed between the OH groups of the gluconic moiety and the polar molecules with in the bacteria cytoplasm and consequently retard all the vital process. And as the new synthesized surfactant was made up from the combination of tow naturally occurring compounds and according to acute toxicity test, this new prepared emulsifier could be used as food additive. As a typical example, the prepared emulsion was used as an egg substitute to produce low-fat mayonnaise, and it had enhanced the properties of mayonnaise.

## Data Availability

The data are available within the manuscript.
